# Medical and Physical Intelligence

**Published:** 1804-04-01

**Authors:** 


### MEDICAL AND PHYSICAL INTELLIGENCE.

### [ FOREIGN AND DOMESTIC. ]

Mr. Buchholz, of Erfurt, recommends the following improved method of preparing arsenic acid: Having mixed together two parts of muriatic acid, of 1,200 specific weight, eight parts of finely pulverised oxyd of arsenic, and twenty-four parts of nitric acid, of 1,250 specific weight; pour the liquor into a glass retort, and let it boil till all the oxyd is dissolved, and no red fumes of the nitrous acid be disengaged. If the nitrous acid should be more concentrated, sixteen parts of it will be sufficient to be gradually added to the above mixture, but then it is necessary to pour this acid to it, during the act of boiling, till the nitrous fumes cease to be disengaged. The liquor is now evaporated to dryness, but care must be taken that no arsenical fumes be evolved. The white dry mass, which is thus obtained, must be preserved in close vessels, either by itself or dissolved in water. The properties of this acid are the following:—It acts as a powerful poison on the animal body. In the dry state it is white and quite tasteless. It is slowly dissolved in from four to six parts of water, of a middle temperature. In boiling water it is soluble in almost all proportions; and even with a quarter part of water, it forms a solution of the consistency of a syrup, which being suffered to stand and cool, deposits crystalline grains. It attracts the moisture of the atmosphere and grows liquid. Its solutions have a sourish taste. In a moderate fire it remains fixed, and becomes a clear and limpid fluid; but by degrees it is changed into oxyd of arsenic, and is volatilised in form of fumes. In the state of being melted, it corrodes the earthen and glass vessels in which it is melting, forming vitreous masses, partly soluble, partly insoluble in water, which depends on the duration of the melting, and the quantity of siliceous earth that is dissolved; these masses being dissolved in water, depose the siliceous earth. It contains in 100 parts $14\frac{1}{2}$ ct. more oxygen than the oxyd of arsenic; or, 100 parts of oxyd of arsenic absorb almost 17 per ct. more oxygen, when changed into arsenic acid.

---

Professor Trommsdorf, of Erfurt, has adopted a new method of preparing muriated barytes, founded on the mutual decomposition of the sulphat of barytes or heavy spar, and of the muriat of lime, which takes place when both are heated and melted together. The experiments made by that gentleman yielded the following results:

1. The heavy spar is decomposed by the muriat of lime in the dry way, but not all at once. 2. The muriat of lime, melted in every every proportion with the sulphated barytes, gives muriat of barytes and some muriat of lime; and if a less proportion of the latter be taken, only a small quantity of sulphated barytes is obtained. 3. The best proportion seems to be, to take equal parts of muriat of lime and of sulphated barytes. 4. The sulphated barytes is entirely decomposed by melting it repeatedly with muriat of lime; and the muriat of barytes, which is thus procured, has a great degree of purity, and may be freed from the adhering muriat of lime by alcohol, or again dissolving and crystallising the mass. 5. The preparation of the muriated barytes, made in this manner, is the most profitable, particularly if the muriat of lime is obtained during other pharmaceutical processes, as the preparation of the spir. sal. ammon. caustic.

The process for obtaining muriated barytes is as follows: Take white sulphated barytes that has been properly glowed, and muriated lime, four pounds of each; pulverise and mix them well, and put them into a large crucible, which is afterwards covered. Keep the vessel over a good fire till it becomes red hot; the mass being melted, pour it into an iron vessel, and let it cool. Reduce the mass into small pieces in an iron mortar, and having poured it into an earthen vessel, infuse it with ten pounds of boiling water, stirring it frequently, and afterwards filtrate it through a strainer of linen. All the liquor having run through, put it aside; then pour the residuum into a clear iron vessel, and dry it over a gentle fire. Mix it again with four pounds of muriat of lime, and put it into the red hot crucible, treating it in the same manner as before. The residuum is dried and treated again with three pounds of muriated lime. All the liquors which are thus obtained, are evaporated in an evaporating dish till a cuticula forms itself on the surface; and in the temperature of 5 to 8° above the freezing point, all the muriated barytes contained in the liquor, will shoot into fine crystals; otherwise the liquor must be farther evaporated till the whole be crystallised. Put the thus obtained crystals on clean blossom paper in a sieve, and place them during several days in an airy warm place. Dissolve them again in water, and let them crystallise; in which manner, crystals of a very white colour will be obtained, if the heavy spar and the muriated lime have been quite free of iron particles, and if the iron vessels that are used in this process be clean and well polished. In this manner from four pounds of heavy spar, three pounds of muriated barytes may be obtained.

---

M. Jean Baptiste-Louis Routier, Physician and Member of the Society of Emulation at Amiens, has lately published at Paris, a pamphlet entitled, Considerations on the Malady peculiar to Women in Labour, called the puerperal fever. This interesting dissertation, particularly so to young students, was the object of a public act in the School of Medicine at Paris; it is divided into three articles: In the first, the author endeavours to prove that the fever fever termed puerperal has no actual existence; that what authors have admitted under this denomination, is a phlegmasia of the order of phlegmasiæ, of the diaphanous or serous membranes, almost always complicated with a fever, which may, says the author, be referred to the orders of known fevers. In the second article, he describes and classes these different complications with which the phlegmasia appears, and by which it is almost always governed; which requires that the physician should turn his whole attention to this part in the malady here referred to. In the third article, M. Routier endeavours to establish, conformably to these views, the treatment proper to the puerperal fever, and the different modes of its termination, &c. &c.

---

A new Work, entitled, An Examination of the Mineralized Remains of Vegetables and Animals of the Antediluvian World, generally termed Extraneous Fossils, illustrated by coloured Plates, by James Parkinson, Part the First, containing the Vegetable Kingdom, will be published on the 1st of June next.

